# Aptamers in Diagnostics and Treatment of Viral Infections

**DOI:** 10.3390/v7020751

**Published:** 2015-02-16

**Authors:** Tomasz Wandtke, Joanna Woźniak, Piotr Kopiński

**Affiliations:** Department of Gene Therapy, Faculty of Medicine, Nicolaus Copernicus University in Toruń, M. Curie-Sklodowskiej 9 St., 85-094 Bydgoszcz, Poland; E-Mails: tomasz_wandtke@wp.pl (T.W.); wozniak.jkw@gmail.com (J.W.)

**Keywords:** aptamer, SELEX, HIV, HCV, H5N1, HSV, HPV, Ebola

## Abstract

Aptamers are *in vitro* selected DNA or RNA molecules that are capable of binding a wide range of nucleic and non-nucleic acid molecules with high affinity and specificity. They have been conducted through the process known as SELEX (Systematic Evolution of Ligands by Exponential Enrichment). It serves to reach specificity and considerable affinity to target molecules, including those of viral origin, both proteins and nucleic acids. Properties of aptamers allow detecting virus infected cells or viruses themselves and make them competitive to monoclonal antibodies. Specific aptamers can be used to interfere in each stage of the viral replication cycle and also inhibit its penetration into cells. Many current studies have reported possible application of aptamers as a treatment or diagnostic tool in viral infections, e.g., HIV (Human Immunodeficiency Virus), HBV (Hepatitis B Virus), HCV (Hepatitis C Virus), SARS (Severe Acute Respiratory Syndrome), H5N1 avian influenza and recently spread Ebola. This review presents current developments of using aptamers in the diagnostics and treatment of viral diseases.

## 1. Introduction

Aptamers are single strand nucleic acid molecules, consisting of DNA or RNA that bind to organic or nonorganic molecules—from single atoms to a wide range of proteins. Aptamers are characterized by high specificity to target molecule and binding affinity: dissociation constant of aptamer/target molecule complex has frequently nanomolar units [1].

**Figure 1:**
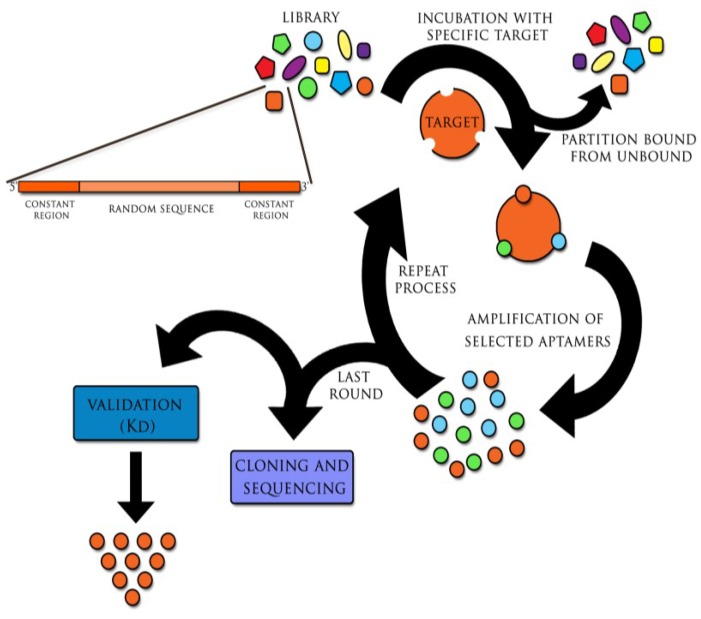
The SELEX (*Systematic Evolution of Ligands by Exponential Enrichment*) process. The initial library of DNA or RNA sequences is incubated with the target molecule. Sequences with the highest specificity and affinity bound to the target, while others are removed from the primary sequences pool. Selected molecules are recovered. Then, if initially DNA molecules were used, they are amplified through polymerase chain reaction (PCR); RNA sequences are reverse transcribed *in vitro* into cDNA and then, similarly as DNA molecules, used in PCR reaction. Amplified oligonucleotides are put through additional selection cycles. Usually 12–15 selection/replication cycles are required to obtain highly specific aptamers. Received aptamers are isolated, cloned and sequenced with following validation so as to find the most specific target aptamer with the highest binding affinity. (Kd, dissociation constant).

Aptamers are generated in the method referred to as SELEX (*Systematic Evolution of Ligands by Exponential Enrichment*). This technique was invented in 1990 independently by two teams: Ellington and Szostak and Tuerk and Gold and has remained immutable since the discovery of aptamers [2,3]. Cycles of selection and replication, followed one by one, are required to obtain target-specific sequences. The combinatorial library, containing all possible sequences with particular length (aptamers have approximately 20–90 nucleotides), is incubated with the target molecule. This process leads to binding with nucleic acids of the highest affinity with the target. At the same time other oligonucleotides are removed from primary sequences pool. Particles of nucleic acids bound with target molecule, undergo recovering and then replication. The process relies on multiple rounds of selection and replication until high specificity and low dissociation aptamers towards the target molecule are generated (Figure 1) [2,3]. Furthermore, certain modifications of SELEX are possible, including its combination with such techniques as capillary electrophoresis (CE) or surface plasmon resonance (SPR). This is expected to reduce the period of selection time, whereas chemical modifications of aptamers’ increase their biostability *in vivo* [4,5,6,7,8,9,10,11,12].

Specific aptamers for single atoms, molecules and also entire bacteria, viruses, cells and tissues can also be engineered by using different selection strategies and modification of incubation conditions (pH, temperature, *etc.*). The secondary and tertiary structures of aptamers provide its binding with the target molecule [13].

Unique properties of aptamers make them competitive to monoclonal antibodies, currently used in conventional laboratory practice. First of all, the time of aptamers selection is relatively short (a few weeks) in comparison to monoclonal antibodies production (a few months). Unquestionable aptamers’ advantage is an opportunity to acquire ligands directed against toxic molecules. It is almost impossible in the case of monoclonal antibodies because of obligatory animals’ immunization. In addition, aptamers are relatively small in size. Thus, it makes them attractive for *in vivo* applications, in comparison to large molecule of monoclonal antibodies. Moreover, aptamers could be easily modified with drugs and immunofluorescence dyes without losing their primary properties [13].

Despite the aptamers have obvious advantages, further clinical studies are necessary before they can be applied in therapy; especially aptamer safety and efficacy should be considered. The first and for the time being the only pharmaceutical aptamer, Macugen (*pegaptanib sodium*), has been admitted in 2004 by the US Agency for Food and Drug Administration (FDA) in therapy of Age-Related Macular Degeneration (AMD) [14]. Several aptamers are currently being evaluated in phases II and III of clinical trials, for example in hemophilia (*ARC19499*) [15], von Willebrand’s disease (*ARC1779*) [16,17] and lung cancer (*AS1411*) [15,18].

Apart from cancers, neurodegenerative, autoimmunological and bacterial diseases, there is currently another significant group of therapeutic targets, *i.e.*, viral infections. However, after decades of research, no treatment of many viral diseases has moved past clinical trials to reach the market or the standard therapy is not satisfactory enough. Thus, aptamers could be potentially used in detection of specific viral molecules and treatment of viral infections [19,20]. This review highlights the current achievements of using aptamers in viral infections.

## 2. Aptamer in the Diagnostics of Viral Infections

Appropriate diagnosis is the key factor for treatment of viral diseases [21]. Nevertheless, viral infections are difficult to distinguish, especially at the onset. If acute infection appears, the patient presents the set of nonspecific signs and symptoms. Time is the most important factor in rapidly developing and epidemiologically dangerous diseases, such as influenza, Ebola and SARS (*Severe Acute Respiratory Syndrome*). On the other hand, chronic viral diseases are asymptomatic or oligosymptomatic. The therapeutic success, focused on organ protection from chronic destruction and failure, e.g., in HIV-1 (*Human Immunodeficiency Virus 1*) or HCV (*Hepatitis C Virus*), depends on early detection of an infective agent.

It has been more than twenty years since aptamers were constructed [2,3]. Up to date, studies on their exploitation in viral diseases are not developed enough. They have been mostly focused on the well-known viruses, such as HIV-1, HCV, HBV (*Hepatitis B Virus*), HPV (*Human Papilloma Virus*), SARS and influenza [22,23,24,25,26,27,28]. Studies on other viruses have not been carried out systematically, e.g., only occasional experiments were conducted with aptamers against Rift Valley Fever, Dengue or different arboviruses [21,29,30].

### 2.1. Experimental Diagnostic Studies with Aptamers and Aptamer-Based Biosensors Conducted on Laboratory Model Samples

A particular attention was focused on influenza diagnosis, due to high risk of infection and remarkable frequency of mutations, resulting in a cyclical appearance of new viral strains with epidemic or even pandemic danger. Most recently, avian influenza was an epidemiological challenge; the disease is characterized by a severe course, high mortality rate and increased risk of zoonotic influenza strain generation. It is capable of moving from one person to another [31,32,33]. Hemagglutinin (HA), a well-known influenza protein, is a glycoprotein expressed in high amounts on the viral surface. It is responsible for fusion of virus with the host cell. There are at least 18 different HA antigens, therefore, it could serve not only for infection diagnosis, but also to distinguish current influenza types and subtypes. Gopinath *et al.* constructed two RNA aptamers, P30-10-16 and A-20, that specifically bind the type A and B of HA, respectively. Aptamers are able to distinguish then influenza type A from type B or even closely related strains of the same influenza subtype [34,35]. It should be emphasized that P30-10-16 binds target molecule (H3N2 of virus A) with more than 15-fold higher affinity, as compared to conventional anti-HA monoclonal antibody [35]. Recently, there were also constructed aptamers able to detect influenza virus type A: H1N1 and H3N2, as well as avian virus, H5N1 [36,37]. Thus, it was shown that aptamers can recognize dangerous influenza strains with high epidemiological risk.

However, it is still necessary to generate simple and available technics in order to increase the benefits of aptamers. Wang and Li generated the construct able to detect avian influenza virus, assigned as aptasensor. The method was based on quartz crystal microbalance (QCM), including attachment of quartz particles to the polymeric, porous hydrogel, containing DNA aptamers [36].

It is thought that similar methods can be used to detect other human infections, such as HIV-1 and Ebola. Tombelli *et al.* generated biosensors based on SPR and QCM technique. They introduced aptamers capable of detecting the HIV-1 Tat protein, using biotin-streptavidin interaction. This approach allows for high specificity distinction between Tat and Rev (other HIV protein, structurally similar to Tat) [24]. Ruslinda *et al.* targeted the same Tat molecule, using biosensor based on the diamond field-effect transistor (FET) technique [23].

Another important field of aptamer application are chronic infections caused by viruses of hepatitis B and C. Aptamer specific for HCV E2 glycoprotein was obtained by Park *et al.* who invented a new diagnostic test—Enzyme Linked Apto-Sorbent Assay (ELASA)—which allowed not only for qualitative analysis, but also quantification of virus particles in the tested samples. Such solution could be used, *i.e.*, for monitoring of antiviral treatment [38].

Liu *et al.* investigated the RNA aptamer (HBs-A22) recognizing HBsAg antigen, present on infected hepatocytes. Thus, it is possible to find HBV infected cells, whereas former assays detected only antigen purified form [25].

An important influence for viral diagnostics development with aptamers have been made by Labib *et al.* who constructed gold microelectrodes with impedimetric properties in order to distinguish biologically active from inactive form of the virus. They applied heat-inactivated Vaccinia as a model. Using specific DNA aptamers they detected, the presence of the viable virus form, since the impedance was lower [39]. Tang *et al.* used the same virus and received DNA aptamer, which was capable of distinguish the infected human cells from their healthy, non-infected counterparts. The idea was based on the presence of unspecified membrane marker appearing on the cell surface [40]. Similar study was carried out by Parekh *et al.*, who additionally defined the target of constructed aptamer as a glycosylated HA presented on infected cells [41].

An important threat to the human health, due to its oncogenic potential, is Human Papilloma Virus. The type 16 of HPV is responsible for approximately 50% of all cervical cancer cases. The direct causative molecule is the viral oncoprotein E7. It is necessary to develop the line of rapid, sensitive, and less costly diagnostic tools, identifying the type of virus infection, as well as a detection of dysplastic endometrial cells with the high risk of malignant transformation. Consequently, HPV is a potential target for aptamers. Toscano-Garibay *et al.* used *in vitro* selection to obtain the RNA aptamer that binds viral E7 oncoprotein. As a part of the biosensor it could provide a powerful diagnostic tool [27]. Graham and Zarbl received fluorescent-conjugated DNA aptamer able to bind superficial determinants of normal cervical epithelium. The cells that initiated malignant transformation did not bind the aptamer [42].

Bruno *et al.* generated DNA aptamers directed against pathogens of the *arbovirus* family, including such viruses, such as Chikungunja, Crimean-Congo Haemorrhagic Fever, Dengue, West Nile and tickborne encephalitis. They received sequences with high specificity and affinity for both the purified, recombinant viral proteins and whole inactive viruses. They proved the value of selected sequences in projected diagnostic methods, *i.e.*, lateral flow chromatographic test strip and fluorescent aptamer-magnetic bead sandwich assay. They also proposed the aptamers to be used for passive immunity and antiviral prophylaxis because of the low immunogenicity. This approach could be promising in any dangerous infection with lack of efficient therapeutic alternative [29].

The spread of Dengue Fever virus is caused by extended occurrence of its host, *Aedes* *aegypti* mosquito, as the result of contemporary climate changes. Fletcher *et al.* constructed three modular biosensors allowing for both qualitative and quantitative analysis of viral infection. It was based on the appearance of fluorescence, while the biosensor has detected the virus. The sensor contained the following modules: (i) sequence complementary to the viral genome, (ii) sequence complementary to the aptamer, (iii) aptamer-EcoRI restriction enzyme complex, (iv) DNA motive bound to fluorescence inhibitor, (v) EcoRI target sequence, and (vi) DNA fragment labeled with a fluorescent dyer (primary fluorescence was quenched by its inhibitor). When aptamer bound the viral genome, the fragment complementary to the aptamer was exposed. The conformational changes of biosensor occurred, EcoRI was released and activated. The enzyme cleaved the DNA sequence stained with fluorescent dyer with subsequent appearance of fluorescence, with intensity proportional to the number of viral copies [21].

Table 1 shows a summary of the described methods.

**Table 1:** Aptamers and aptamer-based biosensors in viral diagnostics. (n/d, no data)

Virus	Aptamer Name	Type	Target	Binding Affinity (K_d_)	Detection Technique	Limit of Detection	Refs.
**Influenza H5N1**	RHA0006 RHA0385	DNA	Hemagglutinin	15.3 nM 24.7 nM	sandwich enzyme linked aptamer assay (ELASA)	0.1 µg/well	[37]
	n/d		surface protein	4.65 nM	QCM-based biosensor coated with the hydrogel	0.0128 HAU	[36]
**HIV-1**	n/d	RNA	Tat protein	1nM	FET-based biosensor	1.2 × 10^9^ molecules	[23]
				n/d	QCM-based biosensor SPR-based biosensor	0.25ppm	[24]
**HCV**	E2-B E2-D	DNA	E2 glycoprotein	4 nM 0.8 nM	enzyme linked apto-sorbent assay (ELASA)	3.13–6.25 × 10^2^ FFU/mL, 16 ng/mL of glycoprotein E2	[38]
**Vaccinia**	n/d	DNA	vaccinia particles	25 nM	AptaVISens-V aptamer-based viability impedimetric sensor	330 PFU	[39]
	PP3		Hemagglutinin	3.24 nM	fluorescence microscope using Alexa Fluor 594-labeled aptamer PP3	n/d	[41]
	TV01		surface protein	7.3 nM	flow cytometry assay using Cy5-labeled aptamer TV01		[40]
**HPV**	13 14 20 28	DNA	epitopes on cell surface proteins of non-infected cells	2.5 nM 7.1 nM 1.6 nM 6.9 nM	confocal microscope	n/d	[42]
	G5α3N.4	RNA	oncoprotein E7	1.9 µM	EMSA assay		[27]
**Chikungunya, Dengue, West Nile**	spectrum of selected aptamers	DNA	viral envelope proteins	spectrum of data	lateral flow chromatographic test strip fluorescent aptamer-magnetic bead sandwich assay	n/d	[29]
**Dengue**	apt_EcoRI	n/d	EcoRI enzyme—one of biosensor modules	n/d	modular biosensor detecting the genetic sequences of Dengue genome	n/d	[21]

### 2.2. Experimental Diagnostic Studies with Aptamer-Based Biosensors Conducted on Natural or Clinical-Based Samples

The usefulness of aptasensors in the detection of viruses was also tested in clinical samples.

Briefly, Bai *et al.* took attempt to combine aptamers with biosensor technology, known as SPR. This model includes the chip coated with both gold layer and streptavidin. DNA aptamers directed against avian influenza H5N1 have been attached to its surface, since they were modified by biotinylation; the material tested in the study was saliva harvested from the poultry. The method does not require any labeling procedures and facilitates quick (1.5 h) detection [19].

Also the invention of new and more cost-effective diagnostic methods enabling early diagnosis of HCV is essential. Lee *et al.* reported the construction of a biosensor utilizing the fluorescent dye (Cyanine3) RNA aptamer directed against HCV core antigen [43], while Chen *et al.* obtained the aptamer that bound viral glycoprotein E2 [26]. These approaches might be crucial for the early diagnosis of hepatitis C at the moment of “window period”, when serum antibodies have not been appeared yet. Commonly, the fluorescent dye-conjugated aptamers seem to be useful in a variety of diagnostic tests.

Table 2 shows more detailed information on the applied solutions.

**Table 2:** Examples of aptamer-based biosensors in experimental diagnostics. (n/d, no data)

Virus	Aptamer Name	Type	Target	Binding Affinity (K_d_)	Detection Technique	Limit of Detection	Sample Type	Refs.
**Influenza H5N1**	n/d	DNA	hemagglutinin	4.65 nM	Spreeta SPR sensing chip	0.128 HAU	poultry swab samples	[19]
**HCV**	ZE2	DNA	glycoprotein E2	1.05 nM	sandwich ELISA	n/d	HCV infected patients’ sera	[26]
	9-14 9-15	RNA	core antigen	142 nM 224 nM	sol-gel chip-based fluorescence assay			[43]

### 2.3. Advantages and Disadvantages of Aptamer-Based Tests in Comparison to Other Diagnostics Methods

The current diagnostic standard for viral infections is Enzyme-Linked Immunosorbent Assay (ELISA) or molecular biology tests [19,21,36,41]. Commonly used ELISA is a multi-stage procedure and is considered to be not efficient enough because of relatively low sensitivity as well as high rate of false positive results [19,21,36]. Moreover, it is difficult in use because of obligatory application of monoclonal antibodies, which are not available for some viral diseases [13].

Most of the currently used immunosorbent tests are able to detect a current disease just at the moment of specific antibody formation directed against the infective agent. However, a specific host immunity develops after several weeks or months from the origin of infection. The problem is known as the so-called “window period”. In addition, some patients receive immunosuppressive therapy and the effective antibody generation may not occur [43,44].

Alternatively, routinely used or even experimental diagnostic molecular tests allow for direct detection of the alien genetic material, without waiting for the immune response of the host. Furthermore, the advantage of molecular assays is their extremely high sensitivity: they are able to detect single viral copies and/or early viral transcripts immediately after the onset of infection [45,46]. Unfortunately, due to very high costs, complicated procedures and necessity of employing highly skilled staff, they have been rarely performed.

Aptamers seem to be an appropriate response to the problems described above. They present an attractive alternative to the currently used procedures, due to their high specificity, affinity of binding to any viral antigen and low cost production. The use of biosensors equipped with the aptamers or another research techniques using these molecules allows for detection of both: early (genetic material, viral proteins) and late (host own antibodies) infection markers [21,23]. Furthermore, aptamers enable to distinguish between infected host cells and not infected ones and may be helpful to recognize active forms of the virus [39,42]. ׳

Table 3 compares a few of the standard diagnostic techniques used in the detection of influenza, HBV and HIV. It should be noted once again that in many cases, the use of currently available diagnostic tools have a number of disadvantages, which could be easily avoided using aptamers [47,48,49,50]. Moreover, the aptamer-based minimum threshold of detection is sometimes lower than in RT-PCR, as in case of influenza virus [36,49]. On the other hand, when the detection threshold is similar, or even higher as compare to currently used techniques, aptamers ensure increased sensitivity and specificity of the diagnostic test [39]. Nevertheless, the time of diagnosis is also significant. As shown in Table 4, the detection time of influenza virus using aptasensor-based technique is remarkable shorter than in other methods [19,47,48,49,50].

**Table 3:** Comparison of clinically used viral diagnostics tests.

Virus	Method	Detection limit	Advantages	Disadvantages	Refs.
Influenza	isolation and identification of the virus	1 EID_50_/mL	sensitivity	time consuming	[47]
	ELISA	1.0 ng	rapid	high rate of false positive results	[48]
	RT-PCR	0.0256 HAU	specificity sensitivity	expensive complicated, highly skilled stuff	[49]
	qRT-PCR	10 copies /reaction			[50]
HBV	ELISA	0.5 pg/mL	as presented above		[51]
	qRT-PCR	18 IU/mL			[52]
HIV	ELISA	0.9–1.2 IU/mL			[53]
	qPCR	18–65 copies/mL			[54]

**Table 4:** Comparison of Avian Influenza Virus detection time with different diagnostic methods.

Method	Virus Isolation	ELISA	RT-PCR	qRT-PCR	SPR Aptasensor
detection time	120–170 h	3 h	5 h	3 h	1.5 h

### 2.4. Future Perspectives of Aptamers in Diagnostic Procedures

Due to the rapid growth of population and different varieties of viruses that are frequently resistant to standard therapeutic treatment, there is clearly an urgent need to develop the new diagnostic methods, characterized by high sensitivity and specificity, allowing for early and rapid pathogen detection.

Biosensor technology is probably the most rapidly growing area of the current diagnosistics of viral diseases. Aptamers are perfect example of molecular recognition biosensor element. The production cost is low, in comparison to monoclonal antibodies. They also provide sensitivity and specificity of the constructed biosensors.

We believe that aptamer-based biosensors could have been applied as promising approach in some specific issues. They could be used for cheap diagnosis at an early stage of the disease, *i.e.*, immediately after exposure to the pathogen, as well as to monitor the treatment process. SELEX versatility and its susceptibility to modifications, as enhancement of the selective pressure, could allow obtaining aptamers detecting precisely the level of viremia, which is below the threshold of the currently used diagnostic methods. Moreover, aptamer-based approach ensures more rapid and cheaper diagnosis.

The aptamer structure might be a major constraint in their future application as diagnostic tool. Many existing aptamers are RNA molecules that are highly susceptible to degradation by nucleases. Consequently, their use as molecular-recognition element of the biosensors may be limited. This problem can be solved by synthesizing a “mirror” analog of these particles that retains their original properties, but are not cleaved by nucleases [7]. Alternative solution includes local modifications of the ribose 2' sites in the aptamer chain [4,6].

In conclusion, we believe that aptamers are molecules potentially attractive for viral diagnostics.

## 3. Aptamers in the Treatment of Viral Infections

**Figure 2:**
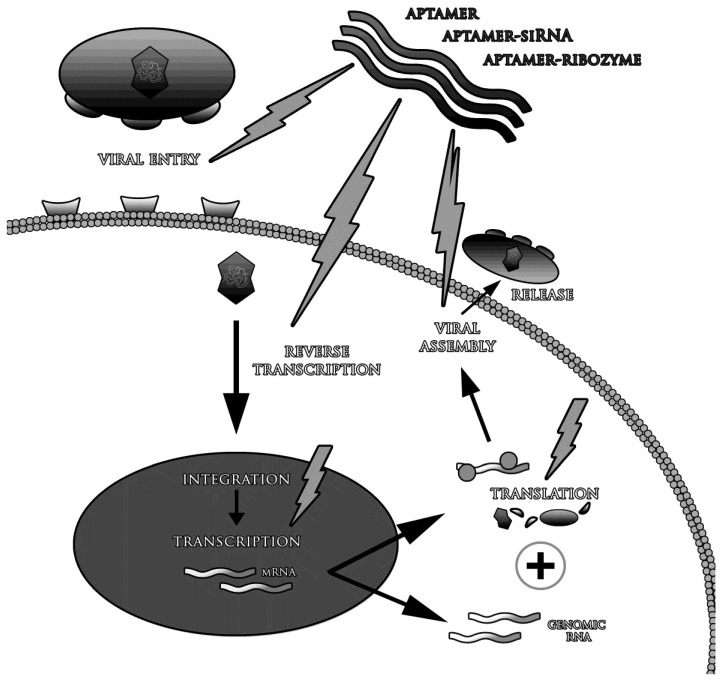
The strategies of antiviral therapy with use of aptamers.

The life-threatening common viral diseases include HCV, HIV-1, SARS, MERS (*Middle East Respiratory Syndrome*) and mentioned above avian flu variants, e.g., H5N1 [31,55,56,57,58,59,60,61,62]. The reason of inefficient medications and vaccines include high virus mutation variability, its low specificity and avoiding the host immune response [55,57,59,62,63,64,65,66,67,68]. It also must be remembered, that many of the existing antiviral drugs, cause side effects and may lead to the development of other diseases than initially treated. They also interact with a variety of medicines, which may weaken or enhance their primary activity [69,70]. Many methods used in the treatment of viral infections have been only partially effective. For example, the standard treatment in HCV (with ribavirin and interferon-alpha) is effective in 50% of cases [71], whereas about 0.5 million of patients die every year [72]. These problems should be the basis for searching new therapeutic tools, more effective and simultaneously less dangerous for patients’. One of the potentially promising solutions might be aptamers directed against any protein of the infected cells and any viral component [25,73]. Aptamers might be used not only to treat the infection, but also to prevent it—viral infection can be inhibited in almost any step of the disease. Many studies confirmed that the most effective therapeutic strategy is to block the penetration of viruses into the cells and/or inhibition of enzymes involved in their replication [74,75]. It is also believed that aptamers are able to selectively stimulate the immune system [76]. They are suitable subjects of structural modifications as *in vivo* biostability improvement and conjugation with other therapeutic molecules, such as small interfering RNA (siRNA) and ribozymes (Figure 2) [77,78,79].

High variable viral genome regions are the common cause of virus resistance to currently used therapies. Thus, there is a requirement to generate aptamers specific to highly conserved nucleic acid regions, where mutations appear relatively rare. The most attention has been paid to HCV and HIV-1 infections, due to their prevalence, severe complications and well-known therapeutic problems. Other viral diseases considered as aptamer targets include influenza, HSV (*Herpes Simplex Virus*) and HBV infections, *i.e.*, the diseases, with commonly occurring immune antiviral response.

Table 5 shows detailed information about experiments described in subsections presented below.

**Table 5:** Aptamers application: *in vitro* therapeutic experiments and models *in vivo*.

Virus	Aptamer Name	Type	Target	Aptamer Application Method	Modification Enhancing Biostability	Inhibitory Effect	K_d_/IC_50_	Refs.
**Influenza H5N1**	A22	DNA	HA	BALB/c mice were intranasally inoculated with the A22 solution	---	>90% decrease in viral loads in mice lungs	n/d	[80]
**Influenza H9N2**	C7-35M	DNA	HA	MDCK-infected culture cells incubated with aptamer	---	inhibition of viral infection in an aptamer-dose dependent manner (1000 pmole inhibits the viral infection by 55%)	n/d	[81]
**Influenza H3N2**	HA12-16	RNA	gHA1	MDCK-infected culture cells incubated with aptamer	none	efficient suppression of viral infection of the cells	n/d	[82]
**HIV-1**	B40, B40t77	RNA	gp120-CCR5	PBMC culture cells incubated with aptamer before infection	2'-fluoro modification	inhibition of viral infectivity (50% at 2 nM)	K_d B40_ = 21 ± 2 nM K_d B40t77_ = 31 ± 2 nM IC_50_ = 2 nM	[79]
	B40t77 iii_4		gp120-CCR5	PBMCs and blood monocyte-derived macrophages (BDMs)- infected cultures incubated with aptamer	inverted thymidine at the 3'-end; dimethoxyltrityloxy-(CH_2_)_6_-SS-(CH_2_)_6_-phospho linker at the 5'-end	inhibition of viral infectivity by 85%	n/d	[83]
	37 NT		HIV-RT	aptamer added to HIV-RT *in vitro* reaction	three 5'-nt and three loop-nt replaced by phosphothionucleosides	reaction rate decreased (100% by 50 nM of aptamer)	K_d_ = 0.66 nM IC_50_ = 2.5 nM	[84]
	DP6-12		Gag protein	293T cells transfected with plasmid encoding aptamer	---	20-fold inhibition of virus production	K_d_ = 130 ± 9 nM	[85]
	Ch A-1 (anti-gp120 aptamer-siRNA chimera)		gp120 (aptamer) tat/rev (siRNA)	RAG-Hu mice were injected with the chimera solution	2'-fluoro modification	reduction in tat/rev mRNA transcript level in mice T lymphocytes between 75% and 90%	n/d	[86]
	anti-gp120 aptamer- siRNA chmiera		gp120 (aptamer) tat/rev, CD4, transportin-3 (siRNA)	RAG-Hu mice were injected intravenously with chimera solution	2'-fluoro modification	significant decrease in viral loads level; stable level of CD4 T lymphocytes	n/d	[87]
	CD4-AsiCs		CD4 (aptamer) gag/vif CCR5 (siRNA)	NSG-BLT mice were administrated intravaginaly with aptamer	none	protection against HIV vaginal transmission	n/d	[88]
**HCV**	ODN 27v	DNA	NSB5	Huh7- JHF1 strain infected cells incubated with aptamer; aptamer enter cells without transfection reagent	none	reduction in virus mRNA levels (90% reduction at aptamer concentrations of 5 µM)	K_d_ = 132.2 ± 20 nM IC_50_ = 196 ± 16 nM	[74]
	B.2	RNA		aptamer added to HCV-NS5B *in vitro* reaction	---	inhibition of NS5B polymerase activity	K_d_ = 1.5 ± 0.2 nM IC_50_ = 10 ± 0.5 nM;	[89]
	NEO-35-s41 G925-s50		NS3	aptamer added to HCV-NS3 protease cleavage and helicase unwinding *in vitro* reactions	---	Inhibition of NS3 helicase and protease activity	protease/helicase NEO-35-s41 IC_50_ = 0.2 µM/20 nM G925-s50 IC_50_ = 0.2 µM/15 nM	[90]
	NEO-III-14U			HeLa-NS3-expressing cells were transfected with aptamer	---	protease activity inhibited in 60%	K_d_ = 4 nM	[91]
	AP30		(-)IRES domain I	aptamer preincubated with template and added to NS5B *in vitro* reaction	---	genetic material replication inhibited by 50%	K_d_ = 36 nM	[92]
**HCMV**	L13	RNA	glycoprotein B	virus particles preincubated with aptamer used to infect HFF cells	2'-amino-modified pyrimidines	infectivity reduction	IC_50_ = 125 ± 20 nM	[93]
	L19		glycoprotein H			100-fold reduction in viral yield blockade of viral entry	IC_50_ = 35 ± 7 nM	
**HSV**	Aptamer-1	RNA	glycoprotein D	virus particles preincubated with aptamer used to infect VERO cells	2'-fluoro modification	blockade of viral entry	K_d_ = 109 nM IC_50_ = 0.8 µM	[75]
**HBV**	S9	RNA	P protein	HepG2.2.15 cells trasfected with plasmid encoding aptamer	---	reduction of replicative intermediates by about 80%–85%	n/d	[94]
**SCV**	ES15	RNA	NsP10	aptamer added to SCV helicase unwinding *in vitro* reaction	---	helicase unwinding activity inhibited in 85%	IC_50_ = 1.2 nM	[95]
**Ebola**	1G8-14 2F11-14	RNA	eVP35 IID	n/d	---	inhibition of EBOV polymerase activity and VP36-nucleoprotein interaction	K_d_ = 30-50 nM	[96]

### 3.1. Blocking of Viral Fusion with the Target Cell

One of the most obvious and frequently studied therapeutic strategies is an inhibition of the viral fusion with the target cell. Viruses penetrate into the cells, using specific surface proteins, which serve as ligands for superficial human molecules. As commonly known, many viruses show a tropism for specific cell types, *i.e.*, HCV, HBV and HIV-1.

HIV-1 glycoprotein gp120, the ligand of T helper cell receptor, CD4, was inhibited by Dey *et al.* who used RNA aptamer B40, and its shorter variant, B40t77. Moreover, they blocked gp120 binding by its T cell co-receptor, CCR5 (*C-C Chemokine Receptor type 5*). They observed decrease in concentration of p24 HIV-1 antigen in supernatants from virus-infected cultures of human PBMCs (*Peripheral Blood Mononuclear Cell*), as measured by ELISA assay [79,97] and number of virus copies in qPCR (*quantitative real-time PCR*) assay [83]. Mutational analysis showed that the aptamer bound to highly conserved region of gp120 [79,83,97].

In HCV infection, E2 glycoprotein was a potential target for aptamer approach. E2 is a co-receptor of human CD81, presented on hepatocytes and B lymphocytes. Chen *et al.* constructed DNA aptamer, assigned as ZE2, competitively blocking E2 in majority of HCV serotypes. Its usefulness was proved in Huh7.5.1, human established cell line of hepatocellular carcinoma. The decrease of both: viral RNA levels in the qRT-PCR (*reverse transcription–qPCR*) analysis and E2 protein concentrations in the Western blot assay were demonstrated [26].

Another application relates to conserved-HA regions of influenza virus. Jeon *et al.* selected A22 DNA aptamer that bound HA specific site, responsible for ligation of human cell receptor. The aptamer-dependent prevention the viral fusion to the target cells was confirmed by *in vitro* cultures in established Madin-Darby Canine Kidney (MDCK) cell line, by measuring the viability of cells after exposure to influenza virus (A/Port Chalmers/1/73). The cells viability increased in the dependent manner to A22 concentration in the cell culture. The best effect was observed between 50 and 100 pmol of A22. The therapeutic potential was also evaluated through an experimental animal model. Mice infected with A/Texas/1/7 influenza strain, threated with aptamer-based drug, tended to lose their weight slower than non-treated control group. Infiltration of mononuclear cells in lungs alveoli was reduced in treated mice after exposition to A22 [80]. Then, Choi *et al.* selected another aptamer, C7-35M, specific for avian influenza virus H9N2 and proved its capacity to inhibit viral infection in MDCK cells in a dose-dependent manner by MTT assay [81]. Cheng *et al.* investigated 24 randomly selected DNA aptamers, from which aptamer 10 strongly bound HA1 subunit of dangerous strain H5N1 avian influenza virus. As it was demonstrated by MTT assay in MDCK cells culture, application of aptamer slowed down the progress of the infection. Authors emphasized that this aptamer application might persuade the host immune system to gain the time for the generation of effective antiviral response [98]. Two other RNA aptamers, assigned as 8-3S and HA12-16, with a similar mechanism of action were recently selected [82,99].

Wang *et al.* adopted the same strategy into human cytomegalovirus (HCMV) infection. They obtained two RNA aptamers (L13 and L19) directed against superficial viral ligand glycoproteins B and H. Aptamers reduced cytomegalovirus infectivity, as it was proven in Human Foreskin Fibroblast (HFF) cell line culture. They were preincubated with aptamers and then exposed to HCMV viral particles. The 50% inhibition of viral plaque formation in culture was observed after administration of 125 ± 20 nM and 35 ± 10 nM of L13 and L19 aptamer, respectively. The authors also found that the binding site of the aptamer have been different from that recognized by specific monoclonal antibody [93].

Aptamer against HSV-1 were designed by Gopinath *et al.* They inhibited viral glycoprotein D, ligand of nectin-1, an actual HSV entry mediator receptor (HVEM). Initially, selected aptamer was too long (113 nt), so it was reduced to 44 nt and modified with 2'-fluoropyrimidines to improve its biostability. Reduced infectious potential of HSV-1 was observed in aptamer dose-dependent manner during plaque formation test in the African green monkey kidney (VERO) cell culture. Selected aptamer had no cytotoxic effects and its IC_50_ (the half maximal inhibitory concentration) was calculated to be 0.8 µM. Importantly, a significant aptamer’s specificity allowed to distinguish HSV-1 and HSV-2 strains [75].

### 3.2. Inhibition of Proteins and Enzymes of Viral Replication Cycle

Another strategy, with the effectiveness comparable to blocking the viral fusion with the cells, is an inhibition of enzymes or other proteins involved in viral replication, transcription and translation.

#### 3.2.1. Blocking of Viral Enzymes with Polymerase Activity

HCV viral protein NS5B (*Nonstructural protein 5B*), RNA-dependent RNA polymerase, is the promising target for aptamer treatment, because of its significance for the virus replication (HCV is a single stranded RNA virus). Biroccio *et al.* selected RNA aptamer B.2., characterized by stem-loop structure, with a specific sequence UAUGGACCAGUGGC recognizing a key element—a GTP binding site of NS5B—responsible for its function. Inhibition of polymerase activity was positively correlated with aptamer concentration, as *in vitro* analysis of the polymerase activity has shown [89]. Bellecave *et al.* obtained DNA aptamer 27v, directed against the same enzyme. It blocked NS5B activity by another way, *i.e.*, by competition the polymerase-binding site with the viral RNA template. Selected aptamer, after its administration to culture cells, reduced the quantity of viral RNA in HCV-permissive human hepatoma cell line (Huh7) infected with HCV JFH1 strain, as it was revealed by qRT-PCR. In comparison to non-aptamer treated cells (7.8 × 106 to 22 × 10^6^ HCV RNA copies), 90%, 68%, and 19% reductions in virus RNA levels at aptamer concentrations of 5 µM, 1 µM, and 100 nM was observed, respectively. Despite the absence of the transfection agent, the aptamer molecules were found intracellularly, as it was proven by confocal microscopy [74].

Feng *et al.* investigated anti-HBV RNA aptamer, designated as S9. The crucial moment for HBV replication and assembly is an interaction of the viral protein R (RNA-dependent RNA polymerase) with stem-loop structure of ε sequence located from the 5' side of the pregenomic RNA. S9 interacted with viral polymerase with high affinity and competed a binding site of viral genetic material. It inhibited HBV replication (reduction of replicative intermediates by about 80%–85%) in human infected cell line (HepG2.2.15), as it was assessed by Southern blot analysis after the cell line transfection with a plasmid vector encoding S9 RNA aptamer [94].

DeStefano and Nair confirmed *in vitro* effectiveness of DNA aptamer, directed against the reverse transcriptase of HIV HXB2 strain. The aptamer, similar to the one mentioned above, competed with natural template for binding site to the enzyme, subsequently inhibiting viral replication. The observation was proved in an *in vitro* primer extension assay. The reaction rate was decreased in 50% and 100% in a presence of 2.5 nM and 50 nM of 37NT aptamer, respectively [84].

#### 3.2.2. Blocking the Activity of Other Enzymes Involved in Viral Replication

Except polymerases, there are other enzymes, which are indirectly involved in virus replication. The best known is NS3 protein (*Nonstructural* *protein3*) consisted of two domains (C- and N-terminal one) with helicase and protease activity, respectively. Both domains are essential for the replication of *Flaviviridae* family, including HCV. Protease domain converts viral proteins necessary for its life cycle, while helicase unwinds DNA and RNA duplexes and allows for replication the genetic material by polymerase. Under physiological conditions, the helicase domain has an affinity to the poly(U) sequence located in the 3'-untranslated region of the viral genome (3'-UTR) [100]. Umehara *et al.* generated a bivalent aptamer with sequences connected by a poly(U) linker. In series of experiments they determined the optimum length of the linker, *i.e.*, 41 and 50 nt. As a result, NEO-35-s41 and G925-S50 aptamers with the highest simultaneous reduction of NS3 both helicase and protease activity have been obtained, as it was confirmed by an *in vitro* enzymatic assays (IC_50_ values for protease and helicase domain inhibition were 0.2 µM/20 nM and 0.2 µM/15 nM for NEO-35-s41 and G925-S50 aptamers, respectively) [90]. In another study, Fukuda *et al.* used ΔNEO-III-14U RNA aptamer, also equipped with a sequence of poly(U), inhibiting the activity of both NS3 protease and helicase domains, as assessed during *in vitro* enzymatic tests and *in vivo* in HeLa cells culture. This effect was significant and aptamer dose-dependent. They hypothesized that the aptamer competed with the 3'-UTR regions of HCV genome for binding site of helicase domain [91].

Another protein necessary for replication and assembly of HCV virions is NS5A (*Nonstructural protein 5A*). NS5A-4 and NS5A-5 aptamers, obtained by Yu *et al.*, allowed for its inactivation in infected Huh7.5 cells; after exposition to anti-NS5A aptamers, viral RNA level was one-fold decrease in comparison to non-treated aptamer controls as assessed by real-time PCR. In particular it was demonstrated that the coupling of NS5A by the aptamer did not allow for new active virion production. It was proved by FFU (the focus forming assay) on naive Huh7.5 cells (six-fold decrease in virus-positive foci was observed in naive cells infected with supernatants harvested from cell cultures treated with aptamers in comparison to non-treated controls). This approach seems to be safe, as anti-viral interferon cell mechanisms remain inactive (mRNA production from interferon’s genes was quantified by qRT-PCR) [101]. Similar observations about the safety profile were noted by Gao *et al.* who found no induction of IFNβ (*Interferon β*), G1P3 and 1-8U genes expression in real-time PCR assay. They obtained NS2-1, NS2-2 and NS2-3 aptamers directed against the NS2 protein (*Nonstructural protein 2*) of the HCV able to effectively inhibit viral replication [102].

One of a few discovered proteins of life-threatening coronavirus SARS is nsP10 (*Nonstructural Protein 10*) enzyme with NTPase/helicase activity. Its function is similar to that of *Flaviviridae* NS3 protein—it unwinds a viral dsDNA [103]. Jang *et al.* targeted nsP10, applying specific RNA aptamer, ES15, which secondary structure contained stem-loop structure with AG repeats. During *in vitro* analysis it inhibited viral enzyme activity in a dose-dependent manner, up to 85% of baseline value (with IC_50_ = 1.2 nM), what was confirmed by fluorescence resonance energy transfer (FRET) test [95].

#### 3.2.3. Blocking the Nucleocapsid Protein of HIV-1

The progress of some viral infections can be prevented by inhibition of nucleocapsid synthesis. Typical therapeutic target is Gag protein of HIV-1 nucleocapsid, because of its low variability, as compared to other sequences of HIV-1 genome. Ramalingam *et al.* used anti-Gag RNA aptamer (DP6-12), in HIV-1 infected cell culture 293T. They observed nearly 20-fold decrease in the number of virions released outside the cells, as it was examined in culture supernatant. DP6-12 aptamer reduced cellular levels of mRNA for Gag, but did not impair the virion release out of the cells what was confirmed by ELISA and Western blot assay. The proper function of the aptamer was based on competition with the packaging signal ψ of HIV-1 for nucleocapsid binding site [85]. Interestingly, in the previous study, Kim *et al.* selected 10-3 anti-HIV-1 RNA aptamer, active in the similar way to DP6-12. It bound two different ψ sequence regions, rich in GC and GU. The circulization of the aptamer to enhance its bio-stability had no effect on binding affinity, what was examined by SPR. Thus, this aptamer seems to be especially promising therapeutic tool [85,104].

### 3.3. Inhibition of Nucleic Acid Sequences Essential for Virus Replication Cycle

The opportunity to select aptamers targeting any molecule, not only protein, makes it possible to use them against viral nucleic acids. Certain regions of their genome interact with proteins responsible for transcription initiation, translation and replication or viral assembly. The generation of aptamers with selective affinity to these regions seems to be promising therapeutic approach.

An Internal Ribosome Entry Site (IRES) of HCV mRNA, involved in viral translation, is a potential attractive therapeutic target, due to its conservative sequence. IRES is composed of four domains, I-IV, located in the 5'-untranslated region (5'-UTR). It is responsible for the initiation of viral replication and mRNA cap-independent translation. IRES binds the small ribosomal subunit (40S) in the host cell, and eukaryotic Translation Initiation Factor 3 (eTIF3). Konno *et al.* applied RNA aptamer AP30, directed against domain I of IRES located at the 3' end of the viral genome antisense strand. AP30 inhibited HCV genetic material replication during *in vitro* analysis by about 50%. Its consensus sequences 5'-UGGAUC-3' and 5'-GAGUAC-3', which were complementary to the SL-E1 and SL-D1 loops in the domain I were responsible for this effect. In this way they prevented attachment of viral RNA polymerase, NS5B, mentioned above [92,105]. Kikuchi *et al.* obtained RNA aptamer containing loop structure with a consensus sequence 5'-UAUGGCU-3', complementary to the loop of the IRES domain II. *In vitro* translation test of IRES-luciferase mRNA confirmed 20%–40% decrease in luciferase activity in presence of 1-17 and 2-02 aptamers [106]. The same team in another study developed aptamer 3-07, directed against the IIId domain of IRES. It successfully inhibited viral *in vitro* IRES-dependent translation and seemed to be much more efficient than the aptamer targeting the second domain of IRES—decrease in the luciferase activity to 10% of the control levels was observed. The inhibiting potential of the selected molecule was also proved in HeLa cells model: cultures transfected with 0.5 pmol 3-07 aptamer showed decrease in luciferase activity up to 45% [107]. Particular attention was focused on simultaneous inhibition of IRES domains II and III-IV; especially, IIId and IIIe regions seem to be crucial in HCV translation. Subsequently, two aptamers, 0207 and 0702, conjugated forms of 2-02 and 3-07, showed a 10-fold stronger binding affinity to the target sequence than any of the components alone; also the IC_50_ value responsible for the same decrease in translational activity was 10-fold lower than 3-07 [108]. Moreover, Romero-Lopez *et al.* combined the activity of hammer head ribozyme (HH363) with properties typical for aptamers. The construct, assigned as HH363-24, bound IRES domain IIId and cleaved the HCV genome in 3' side. It led to simultaneous inhibition of both viral translation and replication. The effectiveness of HH363-24 was proved in Huh7.5.1 human cells, harboring subgenomic RNA replicons derived from HCV-1b. The viral sense strand synthesis was inhibited by nearly 70% as assessed in the qRT-PCR assay. Mutational analysis showed that the inhibitory effect depended not only on typical anti-HCV RNA activity, but on enzymatic ribozyme cleavage activity as well [77].

Some other attempts were focused on aptamers binding to HIV-1 Long Terminal Repeats (LTRs). It is known that LTRs are sequences necessary for proper expression of viral genes; specific aptamers would inhibit the process. Srisawat and Engelke obtained the aptamer with respective activity and its therapeutic effectiveness will be a subject of intensive examination [109].

### 3.4. Delivery of Therapeutic Molecules to Cells Infected with Viruses

An interesting strategy for viral infections is the application of aptamers as specific messengers of oligonucleotides with therapeutic effect, such as small interfering RNA molecules. The use of the construct aptamer-siRNA would limit the specific therapy to the fraction of the target cells, selectively recognized by aptamer. Moreover, it could reduce the side effects accompanying other types of therapy.

Liu *et al.* constructed fluorescein isothiocyanate (FITC)-conjugated RNA aptamer, HBs-A22, specific for a surface antigen HBsAg of HBV infected cells. They detected then infected HepG2.2.15 cells in fluorescence microscopy. They postulated to replace FITC particles with therapeutic agent in the future [25].

Zhou, Neff *et al.* went one step further. They designed a chimeric construct composed of anti-gp120 aptamer and the siRNA molecule directed against the mRNA of HIV-1 tat/rev protein in the model of CHO (Chinese Hamster Ovary cell line) cells. Authors confirmed the binding ability of a fluorescently-labeled aptamer to viral protein. The chimeras penetrated the cells and siRNA molecules appeared intracellularly what was proved in flow cytometry and confocal microscopy. During the second phase, appearance of siRNA molecules inside HIV-NL4-3-infected gp120 expressing CCRF-CEM (Human T cell lymphoblast-like cell line) cells was confirmed by Northern blot. Their therapeutic potential was confirmed by decreased tat/rev mRNA expression in the qRT-PCR reaction. Proposed solution has been extremely beneficial, because anti-gp120 aptamer alone also showed the inhibitory effect on virus infectivity. The therapeutic effect has been therefore strengthened [20,110]. Neff *et al.* renewed the study using humanized mice RAG-Hu (Rag2^−^^/^^−^γc^−^^/^^−^), infected with HIV-1 NL4-3. The chimera (Ch A-1) biostability in mouse serum was improved by 2'-F modification of the aptamer (30% of initial aptamer dose was detectable after 24 h incubation with 50% mice serum). The reduction of viral activity was observed after chimeras application. Authors proved reduction in tat/rev mRNA transcript level in mice T lymphocytes between 75% and 90% by qRT-PCR and also did not find any difference between the percentage of CD4+ T cells in infected mice, as compared to uninfected controls. It consequently proved the protection activity of the constructed chimera [86]. The same *in vivo* model (RAG-Hu; Rag2^−^^/^^−^γc^−^^/^^−^ mice) was used to test anti-gp120 aptamer which was modified at the 3'-end by 2'OMe/2'F 16-nucleotide GC-rich sequence and additional 7-carbon linker. This modification allowed to non-covalent simultaneous binding and transporting three different siRNA molecules, directed against the tat/rev protein, CD4 molecule and transportin-3. The use of this chimera resulted in the lower viral activity as assessed by qPCR assay for total plasma viral loads in infected mice. Moreover, the virus was undetectable after three-weeks of treating in five of eight mice. As observed in previous experiments, construct protected the animals against reduction of CD4 T lymphocytes population [87].

A different strategy was chosen by Zhu *et al.* who converted the anti-CD4 RNA aptamer into the DNA aptamer and combined it with the siRNA molecule directed against the mRNA of HIV-1 protease. Fluorescently labeled aptamer-siRNA conjugates up-take was confirmed by fluorescence microscopy, while inhibition effect was characterized by decrease in mRNA protease expression in CD4+ T cells transfected with pcDNA-HIV-PR plasmid as proved in qRT-PCR assay. Moreover, in comparison to RNA aptamers, their DNA counterparts seem to be more efficient in siRNA delivery [78].

### 3.5. Other Strategies

The aim of another study was to generate the aptamer-siRNA chimeras that could prevent human cells from HIV-1 infection. They used two chimeras: anti-CD4 aptamer/anti-CCR5-siRNA or anti-CD4 aptamer/anti-gag-siRNA [88]. Also Bruno *et al.* signaled the possible effectiveness of aptamers to block Congo hemorrhagic fever, Dengue fever, tick-borne encephalitis and West Nile virus infections [29].

Another approach was proposed by Hwang *et al.* who generated RNA aptamer, CL9, stimulating innate antiviral immunity. It specifically bound a cytosolic receptor RIG-I (*Retinoic* *acid-Inducible* *GeneI*), responsible for foreign molecular pattern recognition in infected cells. It resulted in enhanced antiviral response including the production of IFNβ. According to *in vitro* observations, CL9 aptamer was helpful to prevent cells from invasion, as it was prior applied [76].

## 4. Aptamers against Ebola Infection

Many of the existing viral infections in humans and animals are still considered incurable. Based on recent years’ experiences, we cannot exclude new, dangerous and also life-threatening viruses. The best and most current example is the Ebola virus, which is perceived as severe threat to global public health. It is less than a year since the epidemic of the virus has been spread in the west coast of Africa. Many experiments have been still conducted to discover a vaccine. Enhanced determination to find new therapeutic and diagnostic solutions is necessary.

Up to date, only two reports contribute aptamers as potential tool in Ebola diagnosis and treatment. In 2010, Huang *et al.* investigated aptamers capable of bind zinc-finger antiviral protein responsible for, inter alia, inhibition of Ebola virus replication. Selected aptamers contained conserved sequences “GGGUGG” and “GAGGG” in the loop region, which was important for specific interaction between aptamer and antiviral protein [111]. This information could serve as a base to design molecular diagnostic tests capable to detect Ebola mRNA.

Binning *et al.* used SELEX to obtain aptamer directed against VP35 protein of Ebola virus, one of the most important proteins in the virus replication complex (element of RNA-dependent RNA polymerase). Selected molecule was able to compete with viral dsRNA for binding to VP35 protein and therefore could disrupt interactions between VP35 and Ebola nucleoprotein [96].

## 5. Aptamers—“For” and “against”

Referring to the examples presented in the article, aptamers seem to be effective therapeutic agents that limit amplification of virions or block their penetration into target cells. However, this is only initial success, because most of the presented studies were conducted *in vitro* or in animal models. To fully evaluate the therapeutic potential of these molecules, a few questions about their biostability, pharmacodynamics as well as delivery into cells should be addressed.

### 5.1. The Target Site of Action—Does it Matter? Aptamer vs. siRNA

Considering the therapeutic potential of aptamers, it must be considered that there are at least a few competitors, both low- (e.g., siRNA) and highmolecular weight molecules (e.g., monoclonal antibodies).

Undoubted advantage over siRNA molecules is the interaction between aptamers and a wide spectrum of potential target molecules. The targets of siRNAs are only intracellular RNA particles, while aptamers could also link with molecules located extracellularly, presented in the blood or on the cell surface. Targeting extracellular molecules eliminates the need to involve indirect factors providing aptamer transport through the cell membrane. Although results of many studies showed that utility of aptamers acting in the extracellular space would be sufficient to reduce the extent of viral infection [26,79,82,97], their delivery into target cells, what could give better chance for final curing the infection, still remains a challenge. However, a similar problem occurs in the case of siRNA. The solution might be gene therapy techniques, in particular those associated with viral gene transfer. The effectiveness of this approach has been proven, e.g., in animal models [112,113]. Noteworthy is the study carried out by Bellecave *et al.* in which aptamer was able to penetrate into cells without the involvement of additional carriers. Unfortunately the mechanism of action has still remained unclear [74].

However, gene therapy is actually a branch of experimental medicine, which leaves with more questions than answers. Above all, there is a necessity to proof its safety. It has been shown that viral vectors introduced into cells may lead to the insertional mutagenesis and/or development of disease other than initially treated [114].

On the other hand, widely used monoclonal antibodies are effective only for extracellular targets and cannot penetrate inside the cells.

Therefore, a very interesting solution could be the application of aptamers as highly-specific therapeutic carriers of nucleic acid molecules [115], such as ribozymes or siRNA in HIV experiments conducted by Zhou *et al.* and Neff *et al.* [20,86,87,110]. On the one hand, a high specificity of aptamers allows for the delivery of siRNA into cells that actually require treatment, on the other, siRNA can penetrate into cells by receptor-mediated endocytosis, which solves the problem of using indirect factors ensuring transport of the drug through the cell membrane.

The wide spectrum of target ligands, which is unquestionable advantage of aptamers, makes them competitive to other solutions.

Aptamers associated with the extracellular targets will be the first in clinical practice. For intracellular targets, however, aptamer activity is reduced by their hydrophilic features. Subsequently, it seems to be obvious that the number of necessary analyzes must be carried out to prove the safety of intracellular delivery of a vector encoding aptamers.

It can be predicted that probably the first future clinical trials with aptamers will concern on the viral neutralization in easily accessible areas and organs, *i.e.*, upper respiratory tract, lower airways or female reproductive organs where they will be directed against surface markers of these pathogens. Quite attractive method of application would be an aerosol containing an aptamer, e.g., in the case of influenza virus. An interesting perspective that has been outlined by Wheeler *et al.* is intravaginal application of the gel/cream containing chimeric aptamer/siRNA that might protect against HIV infection [88].

### 5.2. Aptamer’s Stability under Physiological Conditions

No less important factor in the aptamer treatment is their stability under physiological conditions.

Wild-type nucleic acid molecules—DNA, and in particular RNA—are too sensitive for exonucleases activity, to perform a therapeutic function in its natural, unchanged form. Griffin *et al.* consider that the unmodified aptamers directed against targets existing in the blood, may be characterized by half-life time less than 2 min [116].

In order to improve aptamer half-life time, a series of various chemical modifications as it has been highlighted above, can be introduced into their structure. One of the interesting approaches is to invert nucleotide on the 3' side of aptamer. By obtaining two 5' ends, an aptamer becomes resistant to 3'-exonucleases activity, which dominantly occurs in the extracellular environment [117]. Another modifications leading to enhance the stability of aptamers are various changes in the ribose 2' site [4,6,11].

### 5.3. Renal Clearance

Another challenge for aptamer clinical applications, apart from their unsatisfactory biostability, is a very fast renal clearance—the time of aptamers residence in the circulation is too short because of intensive excretion by the renal filtration.

The main factor causing their fast clearance is low molecular weight, usually not exceeding 15 kDa. It should be emphasized that the cut-off threshold of renal filtration is approximately 30–50 kDa. The problem could be solved by conjugation of oligonucleotides with cholesterol molecules or macromolecular polymers, for example polyethylene glycol (PEG) [118,119,120].

There have been no similar studies about antiviral activities of aptamers. However, the results obtained in other experiments, inter alia aptamer tightly binding to blood coagulation factor IXa (half-life was extended from 5 min to 1.5 h), show that this method would be effective [121] in case of antiviral aptamers, as well.

An undoubted advantage of aptamers is, that in contrast to the monoclonal antibodies, they do not lose their primary properties after modification.

### 5.4. Toxicity

Recently, only one aptamer-based drug—Macugen—has entered the clinical trials and gains marketing approval [14]. Therefore, the number of reports on the possible side effects of such products is relatively small.

Other researches have shown that conjugates composed of aptamers and PEG or other high-molecular weight compounds may lead to the infrequent production of neutralizing antibodies directed against the polymers and subsequent rapid filtration of such conjugates by kidneys [122,123,124]. The ability of antisense oligonucleotides and/or therapeutic substance carried by them to accumulate in the interior, particularly of the phagocyte cells was also reported [125].

## 6. Conclusions

Undoubtedly, aptamers are molecules with extraordinary potential. The possibility to obtain a tool specific to any, even unknown, destination target, makes them extremely attractive. It is important that aptamers, which are selected as specific anti-viral molecules, exhibit actual activity in infected cells. However, none of the selected antiviral aptamers entered the phase of clinical trials. There is a real importance to continue the studies. Life-threatening acute viral infections, such as Ebola, H5N1 avian influenza or SARS, are the priority. The same problem consists of dangerous chronic viral diseases, such as HIV, HBV or HCV. The clinical trials are needed to focus on the actual efficacy of antiviral aptamers.

## 7. Executive Summary

### 7.1. Introduction

- Aptamers are single strand nucleic acid molecules, consisted of DNA or RNA, which bind to organic or nonorganic molecules with high specificity and affinity.
- Aptamers are generated in the method referred to as Systematic Evolution of Ligands by Exponential Enrichment (SELEX).
- Properties of aptamers make them competitive to monoclonal antibodies used in conventional laboratory practice.
- The first pharmaceutical aptamer, Macugen (*pegaptanib sodium*) has been admitted by US Agency for Food and Drug Administration (FDA) for the treatment of Age-Related Macular Degeneration (AMD) in 2004.

### 7.2. Aptamers in the Diagnostics of Viral Infections

- The success of treatment in viral diseases depends on the early detection of the infective agent.
- Aptamers allow for detection of both early (viral genes and proteins), and late (antibodies produced by the host) infection markers.
- There are strategies enabling differentiation between infected host cells and uninfected ones.
- Aptamers can differentiate active and inactive virus forms.

### 7.3. Aptamers in the Viral Infections Treatment

- Aptamers are promising solution in viral diseases, if presently used drugs and vaccines are not effective enough. Aptamers can target any element of the virus-infected host cell complex.
- Possible strategies of aptamer application in the treatment of viral diseases include:
  o blockade of the virion penetration into the cells;
  o inhibition of enzymes responsible for viral replication and other crucial processes;
  o conjugation and delivery of therapeutic molecules to virus-infected cells;
  o prevention of infection; and
  o selective activation of the immune system.
